# A Blockchain-Assisted Security Protocol for Group Handover of MTC Devices in 5G Wireless Networks

**DOI:** 10.3390/s24072331

**Published:** 2024-04-06

**Authors:** Ronghao Ma, Jianhong Zhou, Maode Ma

**Affiliations:** 1 School of Computer and Software Engineering, Xihua University, Chengdu 610039, China; 212022085400073@stu.xhu.edu.cn (R.M.); zhoujh@uestc.edu.cn (J.Z.); 2 KINDI Center for Computing Research, College of Engineering, Qatar University, Doha P.O. Box 2713, Qatar

**Keywords:** the fifth-generation cellular network, group handover authentication, MTCD, blockchain

## Abstract

In the realm of the fifth-generation (5G) wireless cellular networks, renowned for their dense connectivity, there lies a substantial facilitation of a myriad of Internet of Things (IoT) applications, which can be supported by the massive machine-type communication (MTC) technique, a fundamental communication framework. In some scenarios, a large number of machine-type communication devices (MTCD) may simultaneously enter the communication coverage of a target base station. However, the current handover mechanism specified by the 3rd Generation Partnership Project (3GPP) Release 16 incurs high signaling overhead within the access and core networks, which may have negative impacts on network efficiency. Additionally, other existing solutions are vulnerable to malicious attacks such as Denial of Service (DoS), Distributed Denial of Service (DDoS) attacks, and the failure of Key Forward Secrecy (KFS). To address this challenge, this paper proposes an efficient and secure handover authentication protocol for a group of MTCDs supported by blockchain technology. This protocol leverages the decentralized nature of blockchain technology and combines it with certificateless aggregate signatures to mutually authenticate the identity of a base station and a group of MTCDs. This approach can reduce signaling overhead and avoid key escrow while significantly lowering the risk associated with single points of failure. Additionally, the protocol protects device anonymity by encrypting device identities with temporary anonymous identity markers with the Elliptic Curve Diffie–Hellman (ECDH) to abandon serial numbers to prevent linkage attacks. The resilience of the proposed protocol against predominant malicious attacks has been rigorously validated through the application of the BAN logic and Scyther tool, underscoring its robust security attributes. Furthermore, compared to the existing solutions, the proposed protocol significantly reduces the authentication cost for a group of MTCDs during handover, while ensuring security, demonstrating commendable efficiency.

## 1. Introduction

Machine-type communication (MTC), also known as machine-to-machine (M2M) communication, has been obtaining increasing attention for the widespread adoption of the fifth generation (5G) wireless cellular networks. With potential to revolutionize a broad spectrum of sectors like healthcare, logistics, manufacturing, process automation, energy, and utilities, MTC stands at the forefront of technological advancement. In certain communication scenarios, such as high-speed trains, convoys, and buses, multiple MTC devices (MTCDs) may move from the coverage area of one base station to another. This occurrence is labeled as a “group handover”, necessitating each MTCD to authenticate during the transition [1].

The 5G wireless network employs a multitude of miniature millimeter-wave cellular base stations, aiming at serving a large number of users. This approach allows for the efficient reuse of limited spectrum resources and supports the access of large-scale MTC devices. Furthermore, adding more base stations can effectively alleviate traffic congestion in wireless channels. Therefore, the 5G wireless network is expected to significantly enhance the performance of wireless connectivity with higher transmission rates, lower communication latency, and greater network capacity. Considering the impending 5G wireless network evolution, a prominent challenge emerges concerning access authentication and data transmission for a vast array of IoT terminals [2]. If each IoT device persists in employing the Extensible Authentication Protocol-Authentication and Key Agreement (EAP-AKA) or the 5G Authentication and Key Agreement (5G-AKA) for its authentication, it would inevitably lead to a surge in signaling and communication overheads [3]. The 5G-AKA protocol is a new authentication and key agreement protocol standardized by the 3rd Generation Partnership Project (3GPP) to be used in the 5G wireless networks. The 5G-AKA protocol plays a pivotal role in enhancing 5G wireless network security by enabling mutual authentication between base stations, core networks, and user devices, thus overcoming the vulnerabilities identified in the 4G wireless networks. It introduces the Subscription Concealed Identifier (SUCI), leveraging encryption to safeguard the sensitive information of users like International Mobile Subscriber Identification (IMSI), against unauthorized interception and malicious base station attacks. This approach significantly bolsters personal privacy and network security, ensuring wireless communications are secure and confidential. Notably, the existing 5G standard, as outlined in the 3GPP standard, exhibits challenges when dealing with concurrent handovers of a cluster of MTCDs. Recent studies [4] have highlighted security loopholes within the handover authentication procedure. These encompass the absence of reciprocal authentication, deficiencies in Key Forwarding Security (KFS), and a heightened vulnerability to Denial of Service (DoS) attacks. Moreover, due to the ultra-dense nature of 5G networks with a larger number of cells, the handover events occur more frequently, leading to increased signaling when a group of MTCDs simultaneously handover from a service base station to a target base station.

Current research has not delivered an efficient and secure solution for MTCD group authentication in 5G wireless networks. Blockchain technology offers decentralization to enhance data security and privacy while shifting authentication from centralized servers to base stations, effectively countering both DoS and Distributed Denial of Service (DDoS) attacks. To address the abovementioned shortcomings, we designed a blockchain-assisted security protocol for group handover (BSPGH) for MTCDs in 5G wireless networks. By uniquely combining blockchain technology with group authentication in 5G wireless networks, the protocol ensures secure handover authentication while remaining simple to deploy with high efficiency. The specific contributions made in this paper can be summarized as follows:

1. We have devised the BSPGH protocol, leveraging the capabilities of blockchain technology. This protocol guarantees the preservation of all security attributes while remaining in alignment with the architecture of the 5G wireless network specified by the 3GPP standard and ensuring its suitability for the MTCD handover scenarios.
2. The proposed BSPGH protocol harnesses the blockchain to establish a decentralized public key management system. It directly culminates in the realization of mutual authentication between base stations and MTCDs. It adeptly streamlines the handover authentication procedure, curtails the volume of interaction messages, safeguards against single points of failure, and fortifies resistance against both DoS and DDoS attacks.
3. The BSPGH solution is built upon the Reliable Malicious KGC-Resistant Certificateless Aggregate Signature (RelCLAS) algorithm and undergoes a formal assessment using the Burrows-Abadi-Needham (BAN) logic and Scyther Tool. It can achieve mutual authentication with key negotiation, anonymity, traceability, perfect forward and backward secrecy, resilience against DoS attacks, and defense against impersonation attacks, etc.

The structure of the remaining paper is as follows. Section 2 provides an overview of the existing handover authentication schemes. Section 3 explains the background knowledge. Section 4 explains the system model and attack model. Section 5 describes the details of the proposed protocol. Section 6 presents the security analysis of the protocol. Section 7 evaluates the performance of the solution. Finally, Section 8 concludes the paper with a summary.

## 2. Related Work

To address the challenges posed by the technologies in 5G wireless networks, the authors in [5] employed aggregated message authentication codes (AMAC) to reduce signaling overheads. By this approach, the group leader aggregates message authentication codes (MACs) from all group members and sends the aggregated information to the network. However, the protocol fails to ensure user privacy because the messages are transmitted in plain text over insecure channels. The solutions in [6,7] need bilinear mapping calculations, which can result in higher computational costs. In [6], a lightweight and efficient group authentication protocol is proposed. This scheme integrates bilinear mapping and aggregate certificateless signature mechanisms to address the real-time secure and efficient access of multiple MTCDs. In [7], a multi-user access authentication scheme has been proposed, which leverages the features of a network architecture combining Mobile Edge Computing (MEC) and Software-Defined Networking (SDN) to perform pre-authentication by predicting a potential target base station for handover. However, this system architecture introduces deployment challenges in practice, and it involves modular exponentiations, which need higher computational costs. In [8], a lightweight group identity authentication scheme is proposed, suitable for both centralized and decentralized settings. It enables all MTCDs to negotiate and generate a group key as a session key for mutual communication. However, this scheme is susceptible to DoS attacks and has high energy consumption when using bilinear pairings. In [1], a privacy-preserving handover authentication protocol suitable for a group of MTCDs in 5G networks is proposed. The protocol aims to reduce signaling costs by aggregating messages from two MTCDs with an aggregated MAC and sending them through an authenticated group member. However, this scheme is susceptible to DDoS attacks.

The solutions in [9,10,11,12,13] all employ blockchain-based techniques for identity authentication. Among them, ref. [9] introduces a blockchain-based protocol to achieve mutual authentication and session key negotiation for vehicles. It is accomplished by introducing an auxiliary blockchain and a parent blockchain, along with the use of an Interplanetary File System (IPFS) to collaborate in information storage. The authentication process by this scheme incorporates elliptic curve cryptography (ECC) and one-way hash functions. The introduction of multiple blockchains may lead to unnecessary resource consumption. Ref. [10] presents a group-based handover authentication scheme for 6G heterogeneous networks, leveraging blockchain for storing authentication information and utilizing aggregate signatures for streamlined batch user authentication. Ref. [11] introduces a collaborative authentication scheme using blockchain in heterogeneous networks. This scheme improves the SM9 algorithm and proposes a verifiable user identity legitimacy through group signature, eliminating data redundancy caused by unfiltered blockchain information in wireless communication. Ref. [12] proposes a lightweight blockchain-based initial and handover authentication protocol for vehicles and infrastructure. This protocol involves vehicles performing lightweight calculations using hash and XOR operations, and the information required for handover authentication is stored in Roadside Units (RSUs) through secure sharing within the consortium blockchain. It can revoke unauthorized vehicles directly using blockchain without the need for a third-party entity. Ref. [13] presents a blockchain-based group key distribution method, distributing and updating group session keys among group members using smart contracts for identity authentication. The authors in [14] have proposed two protocols tailored to different security requirements. However, these protocols are only applicable to scenarios with a predefined trajectory. Ref. [15] proposes a pre-handover authentication mechanism based on the Chinese Remainder Theorem (CRT), allowing user terminals to achieve rapid handover authentication and key negotiation with the target access relay node. However, it is only applicable to fixed trajectory communication in high-speed rail contexts. Ref. [16] produces a group MTCD handover authentication using base stations installed on drones. It applies to extremely specific scenarios and is not suitable for general use. At the same time, there is also the issue of drones used as base stations being unable to sustain long-term energy consumption. The solutions in [5,6,8,10,13,16] are all susceptible to the risk of DoS attacks. During the aggregation of information, the aggregated information can only be successfully verified if all members are legitimate. Attackers can send false aggregate information and intentionally cause the entire group’s verification to fail. Ref. [17] presents a secure and privacy-preserving handover scheme for 5G networks, but it comes with higher computational costs due to the use of multiple modular exponentiation algorithms.

The development of 5G networks has facilitated the support for large-scale connections of MTC devices, offering higher data rates, lower latency, greater connection capacity, and higher energy efficiency. The introduction of large-scale MTC devices enables tens of thousands of devices to be interconnected, which is crucial for applications such as smart cities, smart homes, and industrial automation that require many sensors and actuators to seamlessly connect and communicate. Existing solutions for large-scale MTCD handover authentication in 5G networks have certain security flaws and are almost ineffective in mitigating DoS or DDoS attacks. Moreover, secure functions like bilinear mapping can lead to high computational overheads. All the abovementioned facts motivate us to design a blockchain-assisted group handover authentication protocol to provide sufficient attack prevention, energy efficiency, and fast computation, making it a piece of significant research work. By the BSPGH scheme, the distributed nature of blockchain is utilized to directly achieve mutual authentication between large-scale MTC devices and the target base station, effectively alleviating DoS or DDoS attacks, while also solving the key escrow problem. In terms of resource consumption, the proposed group handover authentication scheme can reduce signaling costs and authentication costs. Our future research will integrate blockchain technology with future communication network standards and protocols to design lightweight protocols.

## 3. Preliminaries

In this section, we introduce some of the technical concepts and cryptographic techniques that will be used in this paper.

### 3.1. RelCLAS

Aggregated signatures are a digital signature technique that efficiently combines *n* independent signatures from *n* users into a single compact signature. This approach allows verifiers to ensure that these *n* users have indeed signed their respective *n* messages, effectively reducing the computational and communication burden during the verification process. In this way, aggregated signatures not only improve data processing efficiency but also optimize resource consumption during storage and transmission. The RelCLAS scheme proposed in [18] is employed in this paper. The RelCLAS scheme typically consists of the following steps:

**Setup:** The AMF and AUSF receive security parameters to generate the system master keys $P_{\mathrm{pub}}$ and $T_{\mathrm{pub}}$, and the system parameters list params is published by the AMF.

**Secret Value Generation:** Users randomly select $msk_{i}\in Z_{q}^{*}$ as the secret value and compute the user’s partial public key $mpk_{i}$.

**Pseudonym Generation:** After receiving the user’s identity identifier ID, AUSF performs an XOR operation on the ID to obtain the anonymous identity TID.

**Partial Secret Key Generation:** AMF randomly selects $r_{i}\in Z_{q}^{*}$ as the secret value and generates the user’s partial public key $R_{i}$ and partial private key $psk_{i}$.

**User Key Generation:** After receiving the partial key from AMF, the user generates the key pairs $\left\{mpk_{i},R_{i}\right\}$ and $\left\{msk_{i},psk_{i}\right\}$.

**Signature Generation:** Each user selects $n_{i}\in Z_{q}^{*}$ to generate the secret value $N_{i}$. Using the parameter list param, some state information, message $M_{i}\in M$ (where M is the message space), their anonymous identity $TID_{i}$, and their private key pair $\left\{msk_{i},psk_{i}\right\}$, they compute the signature $\sigma_{i}$.

**Aggregate:** The aggregate signature generator is the first user entering a new coverage area. It receives signatures from other users and aggregates these signatures to produce the aggregate signature σ.

**Aggregate Verify:** The aggregate signature verifier, i.e., t-gNB, uses the anonymous identities $TID_{i}$ of n users, their corresponding public keys $pk_{i}$, secret values $N_{i}$, the system master key Ppub, and the aggregate signature σ on messages $M_{i},\ldots ,M_{n}$ as input. If the aggregate signature is valid, it outputs true, otherwise, it outputs false.

### 3.2. Blockchain

Blockchain is a collaborative distributed ledger that utilizes multiple computer hosts/nodes in a network to store and manage transaction data. Each host maintains a complete copy of the ledger, eliminating the necessity for a single central authority. Transaction data are organized in chronological order into blocks, with each block containing a certain number of transaction records, typically linked to the previous block utilizing a hash value, forming a chain. It ensures the immutability of transaction data. To ensure the ledger remains consistent across all hosts, the blockchain network uses a consensus algorithm to determine which hosts have the authority to add new blocks. This prevents malicious hosts from tampering with ledger data. Blockchain employs encryption technology to safeguard the confidentiality and integrity of transaction data. Each transaction undergoes a digital signature.

Blockchains are categorized into three types [19]. Public blockchains are open allowing any user to join the blockchain network, view the ledger, and participate in the consensus process. Private blockchains are usually controlled by specific entities or organizations, and only invited participants can join the blockchain network. Consortium blockchains are managed collaboratively by multiple entities or organizations, allowing these participants to share data and jointly manage the blockchain network.

In the system under the study, in normal circumstances, around a small cell controlled by a base station, there are six adjacent cells designated as neighbors. The base station of this cell and those of the adjacent cells are used as nodes in a blockchain network. Each 5G base station in the network serves as a private blockchain node, responsible for storing the public key information of MTCDs. Each base station maintains a complete copy of the blockchain. After an MTCD completes initial registration at a nearby base station, the source base station adds the MTCD’s public key information to the blockchain by creating a new transaction. To reduce storage overhead, each block contains multiple transaction records. These transactions are verified by nodes in the blockchain network and consensus can be achieved with other nodes using the Practical Byzantine Fault Tolerance (PBFT) consensus algorithm. Once a new block is accepted by the other blockchain nodes and added to the blockchain, it is distributed to all nodes, including the target base station, thereby updating their blockchain copies. When an MTCD moves from a location controlled by the source base station to the location controlled by the target base station, during the handover preparation phase, the target base station will search for the MTCD’s public key information on the entire blockchain.

This blockchain consists of numerous blocks, each with a size of 1MB. Each block is stored as a file containing multiple transaction records, each of which includes the public key information of an MTCD. Given that we use public keys based on the elliptic curve secp256k1 with public keys of 256 bits in length according to [20]. Together with other transaction information including transaction inputs, outputs, version, and other fields, and transaction fees, the total comes to approximately 180 Bytes per transaction. Therefore, using Equation (1), we can calculate how many transactions can be stored in a block. Since the size of a block is primarily composed of transaction data, for simplicity, we omit other block information in this calculation.

(1) $$N=\frac{M}{P}$$

where *N* represents the number of users that the base station can serve, *M* is the block size, and *P* is the size of the transaction data; the number of transactions that can be stored reaches into the thousands. Considering that the number of MTCDs under a single base station’s coverage is only in the dozens, the capacity of the blockchain to store MTCD public key information far exceeds the needs of a group of MTCDs.

## 4. System Background

### 4.1. System Model

**Figure 1:**
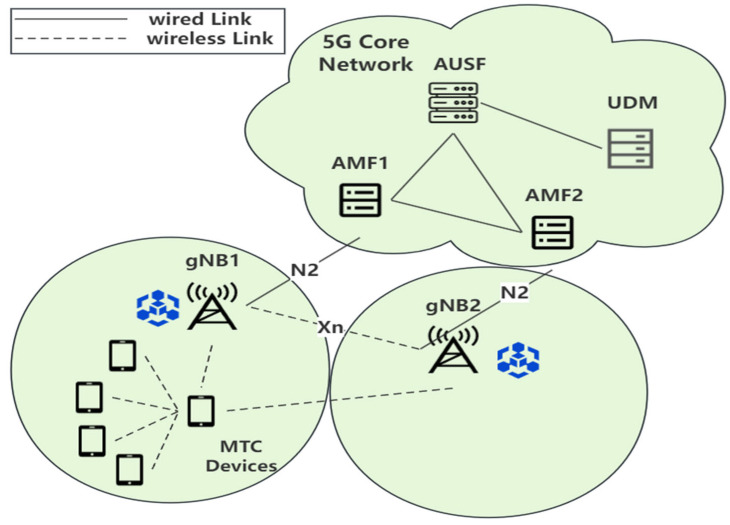
System model.

The system under study follows the structure of the 5G wireless cellular network specified in the 3GPP TS 23.501 R16 [21]. As depicted in Figure 1, the 5G wireless network consists of a core network (CN) and radio access networks (RANs). The devices involved in the CN mainly include the Access and Mobility Management Function (AMF), Authentication Server Function (AUSF), and Unified Data Management (UDM), while Next Generation Node B (gNB) and user equipment or MTCDs exist in the RANs. The entities primarily involved in the handover process are MTCDs, gNB, AMF, AUSF, and UDM.

**AMF:** AMF is responsible for managing access and mobility-related tasks of user devices, ensuring network efficiency, security, and reliability. In the system, in our group handover authentication phase, the AMF does not need to forward authentication information anymore.

**AUSF:** AUSF is responsible for handling authentication and security-related tasks for MTCDs. It verifies the identity of MTCDs and the security credentials they provide. In our system, the AUSF primarily generates anonymous identities for both MTCDs and gNBs.

**UDM:** UDM is responsible for managing and accessing user data. In our system, the UDM stores the permanent identity identifiers of MTCDs and gNBs.

**MTCD:** In our system, an MTCD, which is a user in the 5G network, initially needs to send a request for registering its identity to the AMF. The MTCD undergoes identity authentication with the gNB upon accessing the network.

**gNB:** gNB is the base station in the 5G wireless network. It is responsible for MTCD’s access and connection, as well as reasonably allocating wireless communication resources.

**Blockchain:** Blockchain is a distributed database that ensures secure storage and sharing of data by creating a continuously growing and tamper-resistant chain of data records. In our system, a private blockchain is utilized as the secure data structure consisting of registered MTCDs. Each gNB maintains a backup copy of the blockchain.

In the 5G wireless networks specified by the 3GPP standards, when an MTCD enters the signal range of a gNB, it sends an authentication request to the gNB. This request is then forwarded by the gNB to the AMF, which is in turn forwarded to the AUSF. After the AUSF retrieves the necessary key information from the UDM, it executes the authentication process and returns the authentication response to the MTCD through the AMF and the gNB, completing further authentication procedures. In a typical 5G core network, one AUSF typically serves multiple AMFs, and each AMF manages connections with multiple gNBs. By the proposed scheme, decentralization of the authentication entities is achieved by integrating blockchain technology with the 5G system model specified by the 3GPP standard. In this system, each gNB is part of a private blockchain network and holds a copy of the entire blockchain. Once an MTCD completes the initial registration, there is no need to forward the authentication requests to the AUSF and UDM again. When an MTCD needs to undergo a handover authentication, the AMF only needs to forward the information of the service-gNB (s-gNB) to the target-gNB (t-gNB). At this point, the gNB can directly verify the identity of the MTCD using the information stored on the blockchain, without further forwarding to the AMF, AUSF, or UDM. To simplify the design, the proposed scheme focuses on the most common scenario, where all MTCDs connect to the gNB in their home network via 3GPP standard access technologies. Moreover, the connection between the gNB and the 5G core network is provided by a wired connection that is safeguarded by an Internet Protocol Security (IPSec) tunnel. If the MTCD and the gNB successfully achieve mutual authentication, the MTCD can be considered to have secure access to the legitimate 5G network.

In a cellular network architecture, to effectively reuse frequency resources, the entire service area is divided into numerous cells shaped as regular polygons, such as hexagons. Consequently, around a cell controlled by a gNB, typically six adjacent cells are designated as neighbors. During a handover, an MTCD can only hand over to one of these six neighboring cells, which is the cell controlled by the t-gNB associated with the current cell controlled by the s-gNB [22].

### 4.2. Attack Model

The attack model for the network under study is the Dolev–Yao model [23], by which attackers are assumed to be rational, powerful, and fully controlled entities capable of intercepting, tampering with, and sending messages. They can also attempt to break the security function of the protocol by analyzing communication content. In the RAN domain of the 5G network, there are security vulnerabilities in the wireless communication between MTCDs and gNBs, making them prone to malicious attacks such as information interception and tampering. According to the specification [21], the N2 interface employs IPsec and IKEv2 certificates to protect communications, ensuring their integrity and confidentiality, and preventing replay attacks. Therefore, the connection between the 5G core network and the gNB is considered secure. However, the connection between MTCDs and gNBs is weaker and potentially insecure. It is assumed that the interior of the 5G core network and its network functions are secure, ensuring the safety of connections between network functions. In contrast, other entities in the RAN are not completely trusted.

Given these considerations, an ideal 5G identity handover authentication protocol should support security features including device anonymity, bidirectional identity verification, secure data transmission, and perfect forward secrecy. Additionally, it should be capable of defending against active attacks, including impersonation, linkability attacks, replay attacks, man-in-the-middle attacks, and DoS attacks, as well as passive attacks like eavesdropping and location tracking.

## 5. The Proposed BSPGH

The details of the BSPGH protocol are presented in this section. By combining group authentication with blockchain technology, the BSPGH scheme demonstrates various security attributes. The BSPGH scheme operates in five distinct phases including system initialization and registration, handover preparation, first MTCD handover authentication, group handover authentication, and connection establishment. The notations used are shown in Table 1.

### 5.1. System Initialization and Registration

**Figure 2:**
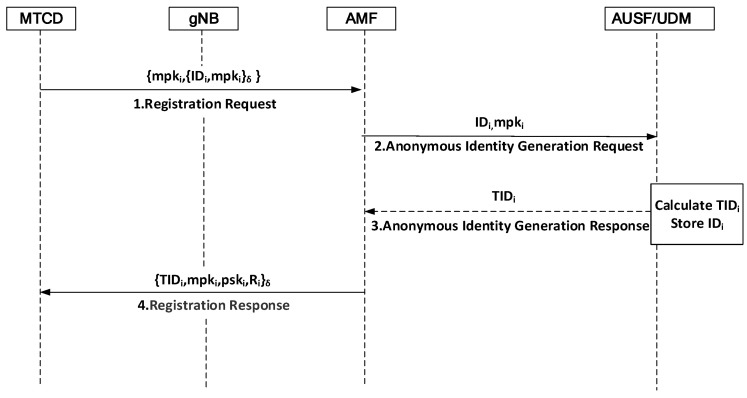
Initial registration.

(1) System initialization: Given security parameter $1^{K}$, AMF selects a large prime number q and $E(F_{q})$ as the elliptic curve over a finite field $F_{q}$. Let G be a cyclic additive group generated by generator P with order q. $H_{0}=Z_{q}\times {\left\{0,1\right\}}^{*}\to Z_{q}$, $H_{1}:G\times {\left\{0,1\right\}}^{*}\to Z_{q}$, $H_{2}:{\left\{0,1\right\}}^{*}\times G\times {\left\{0,1\right\}}^{*}\times G\times G\to Z_{q}$, $H_{3}:{\left\{0,1\right\}}^{*}\times {\left\{0,1\right\}}^{*}\times {\left\{0,1\right\}}^{*}\times {\left\{0,1\right\}}^{*}\times G\times G\to Z_{q}$ and $H_{4}:{\left\{0,1\right\}}^{*}\times {\left\{0,1\right\}}^{*}\times G\to Z_{q}$ are hash functions. AUSF chooses a random element $\alpha \in Z_{q}$ and calculates the corresponding public key $P_{pub}=\alpha \cdot P$. AMF chooses a random element $\beta \in Z_{q}$ and calculates the corresponding public key $T_{pub}=\beta \cdot P$. Finally, the AMF publishes the system parameter $params=\left(P,q,G,P_{pub},T_{pub},H_{0},H_{1},H_{2},H_{3},H_{4}\right)$.
(2) Initial registration: To protect identity privacy, each MTCD and gNB should first register with the AUSF to obtain their own pseudonyms. Below, we use the MTCD with the real identity $ID_{i}$ as an example to explain the specific registration process, which is shown in Figure 2.
  $$Step\text{-}1:MTCD\to AMF:\{mpk_{i},\{ID_{i},mpk_{i}{\}}_{\delta }\}$$

The MTCD generates a secret value and a corresponding partial public key. It randomly selects a random number $msk_{i}\in Z_{q}$ as its secret value and calculates the partial public key $mpk_{i}=msk_{i}\cdot P$. Then, it computes $\delta =H_{0}\left(msk_{i}\cdot T_{pub}\right)$ as the symmetric encryption key and sends its identity $ID_{i}$ in a message to AMF.

$$Step\text{-}2:AMF\to AUSF/UDM:\{ID_{i},mpk_{i}\}$$

AMF checks the identity of MTCD and forwards that identity and the public key to AUSF. The anonymous identity ID is generated by the AUSF, while the UDM stores the permanent identity ID.

$$Step\text{-}3:AUSF/UDM\to AMF:\{TID_{i}\}$$

Upon receiving $ID_{i}$, AUSF calculates the temporary anonymous identity $TID=ID⊕H_{1}\left(\alpha \cdot mpk_{i}\right)$. AUSF then forwards $TID_{i}$ to AMF. Simultaneously, AUSF also forwards the identity $ID_{i}$ to UDM, which is responsible for storing $ID_{i}$.

$$Step\text{-}4:AMF\to MTCD:\{TID_{i},mpk_{i},psk_{i},R_{i}{\}}_{\delta \;}$$

AMF generates the secret value and a corresponding partial public key for MTCD. It randomly selects a random number $r_{i}\in Z_{q}$, calculates $R_{i}=r_{i}\cdot P$, $h_{i1}=H_{2}\left(TID_{i},mpk_{i},R_{i},T_{pub}\right)$, and $psk_{i}=r_{i}+\beta \cdot h_{i1}$, then computes $\delta =H_{0}\left(\beta \cdot mpk_{i}\right)$. After forwarding the message to $MTCD_{i}$, $MTCD_{i}$ stores $TID_{i}$, calculates $h_{i1}$, and verifies that $psk_{i}\cdot p=R_{i}+h_{i1}\cdot T_{pub}$. It then sets $PK_{i}=\{mpk_{i},R_{i}\}$ and $SK_{i}=\{msk_{i},psk_{i}\}$ as the public–private key pair. The AMF sends the public key to the gNB, and the gNB uploads the public key pair to the blockchain.

(3) Initial authentication: All MTCDs, the AUSF, and the UDM perform the initial authentication following the 5G-AKA scheme. The gNB and AMF monitor the movement trajectory of each MTCD to determine if some MTCDs could form a group based on the grouping algorithm described in [24] that supports the mathematical correlation required to form an MTCD group. If such a correlation is found, these MTCDs will be considered as a group.

### 5.2. Handover Preparation

**Figure 3:**
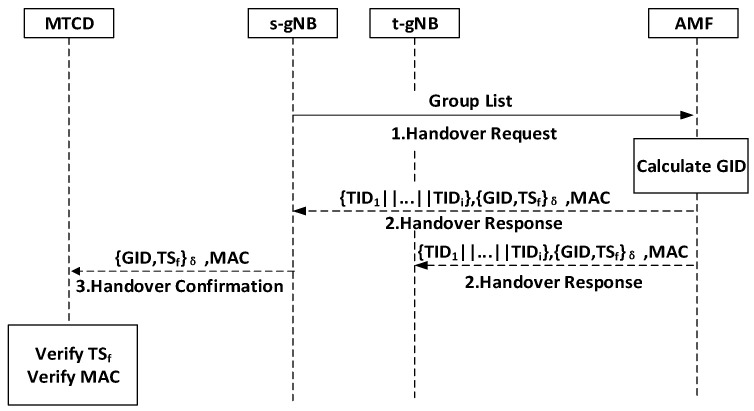
Handover preparation.

This phase occurs before the handover, preparing the necessary key materials for the first MTCD handover authentication and group handover authentication. This phase is shown in Figure 3.

$$Step\text{-}1:s\text{-}gNB\to AMF:\{TID_{s}\}$$

The s-gNB sends a handover request to the AMF containing the neighboring gNB list and the temporary identity identifiers TIDs of all group members.

$$Step\text{-}2:AMF\to s\text{-}gNB/t\text{-}gNB:{\left\{\{{TID}_{1}||\ldots |{|TID}_{i}\},\{GID,{TS}_{f}\right\}}_{\delta },MAC\}$$

After receiving all TIDs from the group members, the AMF computes the group key $GID=H\left(\sum_{i=1}^{n}TID\right)$. The AMF encrypts the timestamp $TS_{f}$ and generates the MAC as $MAC=H\left(GID|{|TS}_{f}\right)$. This message is then broadcasted to the base stations. Upon receiving the message, the t-gNB queries the public key information of MTCDs stored in the blockchain within the group and stores the queried information locally.

$$Step\text{-}3:s\text{-}gNB\to MTCD:\{{GID,{TS}_{f}\}}_{\delta },MAC\}$$

s-gNB broadcasts the message to all MTCDs within the group. Each MTCD decrypts the message to obtain the GID and verifies the received message’s timestamps and MAC to determine its authenticity.

### 5.3. First MTCD Handover Authentication

**Figure 4:**
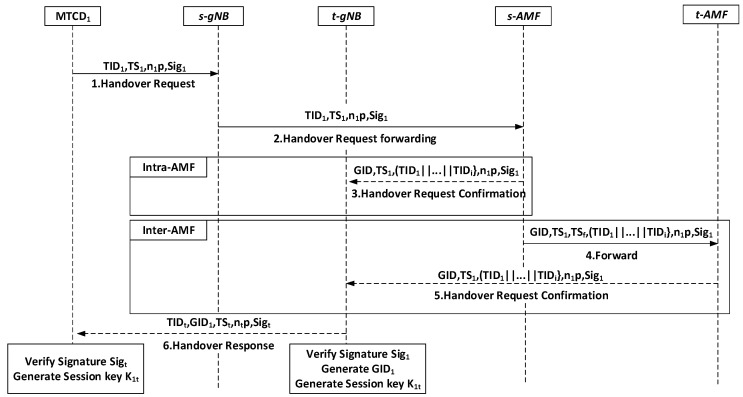
First MTCD handover authentication.

This phase involves one handover authentication that occurs when the first MTCD enters the range of the t-gNB. After the handover authentication of the first MTCD is successful, subsequent group handover authentication processes will be carried out. This phase is shown in Figure 4.

$$Step\text{-}1:MTCD\to s\text{-}gNB:\{{TID}_{1},{TS}_{1},n_{1}\cdot P,{Sig}_{1}\}$$

When the first MTCD enters the coverage range of t-gNB within the group, $MTCD_{1}$ selects a random number $n_{1}\in Z_{q}$ and computes its signature as $Sig_{1}=h_{13}msk_{1}+n_{1}+h_{12}psk_{1}$. Where $h_{12}=H_{3}\left(TID_{1},TS_{1},PK_{1},n_{1}\cdot P\right)$ and $h_{13}=H_{4}\left(TID_{1},n_{1}\cdot P\right)$. Afterwards, ${MTCD}_{1}$ sends its $TID_{1}$, timestamp $TS_{1}$ and $n_{1}\cdot P$, and $Sig_{1}$ to s-gNB.

$$Step\text{-}2:s\text{-}gNB\to s\text{-}AMF:\{{TID}_{1},{TS}_{1},n_{1}\cdot p,{Sig}_{1}\}$$

s-gNB checks the timestamp in the received message and forwards the message to s-AMF.

$$Step\text{-}3:s\text{-}AMF\to t\text{-}gNB:\{GID,{TS}_{1},({TID}_{1}||\ldots ||{TID}_{i},n_{1}\cdot P,{Sig}_{1}\}$$

When the handover authentication occurs within the same AMF, after receiving the handover request, s-AMF responds by sending the temporary identity identifiers $TID_{1}\ldots TID_{i}$ of all group members, along with the GID as the group key material, and forwards $n_{1}\cdot P$ and ${Sig}_{1}$ to t-gNB. And after receiving the handover response from s-AMF, t-gNB queries the public key of $MTCD_{1}$ on the blockchain and calculates $h_{11}=H_{2}\left(TID_{1},mpk_{1},R_{1},T_{pub}\right)$, $h_{12}=H_{3}\left(TID_{1},TS_{1},PK_{1},n_{1}\cdot P\right),$ and $h_{13}=H_{4}\left(TID_{1},n_{1}\cdot P\right)$. It verifies the signature $Sig_{1}\cdot P=h_{13}\cdot mpk_{1}+n_{1}\cdot P+h_{12}\cdot R_{1}+h_{11}\cdot h_{12}\cdot T_{pub}$, and if the equation holds, $MTCD_{1}$’s signature is successfully verified. t-gNB then selects a random number $n_{t}\in Z_{q}$ and generates the session key $K_{1t}=n_{1}\cdot n_{t}\cdot P$. It also creates the signature $Sig_{t}=H_{0}\left(GID,{TID}_{t},TS_{t},n_{t}\cdot P\right)$ and computes $GID_{1}=\sum_{i=2}^{n}H\left(TID_{i}\right)$ as the group key material to authenticate $MTCD_{1}$. It then sends a message to $MTCD_{1}$.

$$Step\text{-}4:s\text{-}AMF\to t\text{-}AMF:\{GID,{TS}_{1},{TS}_{f},({TID}_{1}||\ldots ||{TID}_{i},n_{1}\cdot P,{Sig}_{1}\}$$

When the handover authentication occurs between different AMFs, the s-AMF forwards the message to the t-AMF.

$$Step\text{-}5:t\text{-}AMF\to t\text{-}gNB:\{GID,{TS}_{1},({TID}_{1}||\ldots ||{TID}_{i},n_{1}\cdot P,{Sig}_{1}\}$$

Similarly, upon receiving the forwarded handover request, the t-AMF sends the temporary identity identifiers ${TID}_{1}\ldots {TID}_{i}$ of all group members, along with the GID as group key material, in response. It then forwards $n_{1}\cdot P$ and ${Sig}_{1}$ to t-gNB. Upon receiving the message, the t-gNB proceeds with the verification.

$$Step\text{-}6:t\text{-}gNB\to MTCD:\{{TID}_{t},{GID}_{1},{TS}_{t},n_{t}\cdot P,Sig_{t}\}$$

After receiving the message from t-gNB, $MTCD_{1}$ generates the signature $Sig_{t}^{\prime }=H_{0}\left(GID,{TID}_{t},TS_{t},n_{t}\cdot P\right)$. If $Sig_{t}^{\prime }=Sig_{t}$, the identity verification of t-gNB is successful. Then, $MTCD_{1}$ generates the session key $K_{1t}=n_{1}\cdot n_{t}\cdot P$. At this point, the mutual authentication and key negotiation between $MTCD_{1}$ and t-gNB are complete.

### 5.4. Group Handover Authentication

$MTCD_{1}$ initiates an aggregated signature request to other group members in the group, broadcasting the temporary identity $TID_{t}$ of the t-gNB. $MTCD_{1}$ also sends its temporary identity $TID_{1}$ and the group key material $GID_{1}$. Other members within the group verify the identity of $MTCD_{1}$ by validating $GID=GID_{1}+H\left(TID_{1}\right)$.

$$Step\text{-}1:{MTCD}_{1}\to {MTCD}_{i}:\{{GID}_{1},{TID}_{t},{TID}_{1},{TS}_{1}\}$$

$$Step\text{-}2:{MTCD}_{i}\to {MTCD}_{1}:\{{TID}_{i},{TS}_{i},n_{i}\cdot P,Sig_{i}\}$$

The group member $MTCD_{i}$ receives the aggregated signature request, verifies the identity of $MTCD_{1}$, selects a random number $n_{i}\in Z_{q}$, generates its respective signatures, and sends its signature $Sig_{i}=h_{i3}msk_{i}+n_{i}+h_{i2}psk_{i}$.

$$Step\text{-}3:{MTCD}_{1}\to t\text{-}gNB:\{({TID}_{2}||\ldots ||{TID}_{i}),(n_{2}\cdot P||\ldots ||n_{i}\cdot P),Sig,{TS}_{i}\}$$

After receiving the signatures from all group members, $MTCD_{1}$ calculates the aggregated signature $Sig=\sum_{i=2}^{n}Sig_{i}$ for the group and sends the aggregated signature to the t-gNB for performing group signature verification using the equation $\sum_{i=2}^{n}{Sig}_{i}\cdot P=\sum_{i=2}^{n}h_{i3}\cdot {mpk}_{i}+\sum_{i=2}^{n}n_{i}\;\cdot P+\sum_{i=2}^{n}h_{i2}\cdot R_{i}+\sum_{i=2}^{n}h_{i1}\cdot h_{i2}\cdot T_{pub}$ to pre-authenticate all group members. If the equation holds, the t-gNB verifies all MTCDs. Subsequently, t-gNB generates the session key $K_{it}=n_{i}\cdot n_{t}\cdot P$ between $MTCD_{i}$ and the t-gNB.

$$Step\text{-}4:t\text{-}gNB\to {MTCD}_{1}:\{{TID}_{t},{TS}_{t},n_{t}\cdot P,Sig_{t}\}$$

The t-gNB verifies the signatures of the group members to authenticate their identities. If the identity authentication is successful, the t-gNB sends the timestamp $TS_{t}$, random number $n_{t}\cdot P$, and its own signature $Sig_{t}=H_{0}\left(GID,TID_{t},TS_{t},n_{t}\cdot P\right)$ to the $MTCD_{1}$, because it has passed the first MTCD handover authentication already.

$$Step\text{-}5:{MTCD}_{1}\to {MTCD}_{i}:\{{TID}_{t},{TS}_{1},{{TS}_{t},n}_{t}\cdot P,Sig_{t}\}$$

**Figure 5:**
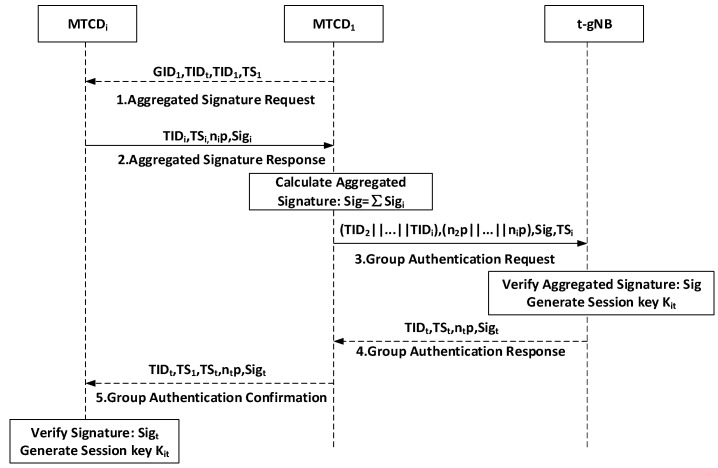
Group handover authentication.

After receiving the message from $MTCD_{1}$, $MTCD_{i}$ generates the signature $Sig_{t}^{\prime }=H_{0}\left(GID,TID_{t},TS_{t},n_{t}\cdot P\right)$. If $Sig_{t}^{\prime }=Sig_{t}$, the identity verification of t-gNB is successful. If the verification is successful, $MTCD_{i}$ generates the session key $K_{it}=n_{i}\cdot n_{t}\cdot P$ between $MTCD_{i}$ and the t-gNB. At this point, mutual authentication and key negotiation between $MTCD_{i}$ and t-gNB are complete. This phase is shown in Figure 5.

## 6. Security Evaluation

In this section, we first prove the logic correctness of the proposed BSPGH scheme by using BAN logic and perform a formal verification of the security functionality of the BSPGH protocol by using the Scyther. Furthermore, a security analysis is conducted to identify security properties held by the BSPGH protocol and its robustness against various malicious attacks is described. The results can provide insights into the protocol’s effectiveness and capacity to resist various threats.

### 6.1. Formal Proof by BAN Logic

BAN logic is an important method for the formal analysis of security protocols [25], aiming to verify their security and correctness. The fundamental principle of BAN logic is to establish a set of rigorous logical rules to describe the semantics of entities, message exchange, and information states involved in the protocol. To apply BAN logic to prove the BSPGH protocol, we formalize the protocol into an idealized form. We then propose assumptions and objectives and use BAN logic symbols and rules for derivation, such as message meaning rules, temporary value validation rules, arbitration rules, belief rules, and reception rules. By manually applying the derivation rules, we aim to achieve the objectives and verify the security properties of the protocol.

The rules of BAN logic for derivation can be described as follows:

(R1) The Message Meaning Rule: $\frac{P|\equiv Q\overset{K}{⟷}P,P◃X_{K}}{P\left|\equiv Q\right|∼X}$, $\frac{P|\equiv \overset{K}{\to }Q,P◃X_{K^{-1}}}{P|\equiv Q∼X}$, $\frac{P|\equiv P\overset{Y}{⇌}Q,P◃X_{Y}}{P|\equiv Q∼X}$. The first means that if party P trusts that K is a shared key between P and Q, and if P has received a message X encrypted with K before, then P believes that Q has sent the message X. The second means that if P believes that user Q’s public key is K, and P sees that the message X, which is signed with Q’s private key, is $K^{-1}$, then P believes that the message X was sent by Q. The third means describes the shared secret.

(R2) The Freshness Rule: $\frac{P|\equiv \#(X)}{P|\equiv \#(X,Y)}$. This rule means that if one part of the message is fresh, then the entire message is fresh.

(R3) The Nonce Verification Rule: $\frac{P|\equiv \#(X),P|\equiv Q|∼X}{P\left|\equiv Q\right|\equiv X}$. This rule means that if P believes that message X is fresh and believes that Q has sent X before, then P believes that Q believes X.

(R4) The Belief Conjunction Rule: $\frac{P\left|\equiv Q\right|\equiv \left(X,Y\right)}{P\left|\equiv Q\right|\equiv X}$, $\frac{P\left|\equiv X,P\right|\equiv Y}{P|\equiv (X,Y)}$, $\frac{P|\equiv (X,Y)}{P|\equiv X}$. This rule means that if P believes that party Q believes messages X and Y, then P believes that Q believes X.

(R5) The Jurisdiction Rule: $\frac{P\left|\equiv Q\right|\Rightarrow X,P\left|\equiv Q\right|\equiv X}{P|\equiv X}$. This rule means that if P believes Q has jurisdiction on message X, and P believes Q believes X, then P believes X.

#### 6.1.1. Formalized Protocol

To idealize the protocol, we describe the messages in the proposed protocol as follows:

**Messages-1**: The s-gNB sends $\left\{TIDs\right\}$ to AMF.

(M1) AMF◃TIDs

**Messages-2**: The AMF sends $\left\{(TID_{1}||\ldots ||TID_{i}),{\left\{GID,TS_{f}\right\}}_{\delta },MAC\right\}$ to s-gNB.

(M2) $s\text{-}gNB◃\{\{TID_{1}||\ldots ||TID_{i}\},{\left\{GID,TS_{f}\right\}}_{\delta },MAC\}$

**Messages-3**: The s-gNB sends ${\left\{GID,TS_{f}\right\}}_{\delta },MAC$ to MTCD.

(M3) $MTCD◃\{{\left\{GID,TS_{f}\right\}}_{\delta },MAC\}$

**Messages-4**: The $MTCD_{1}$ sends $\left\{TID_{1},TS_{1},n_{1}\cdot p,Sig_{1}\right\}$ to s-gNB.

(M4) $s\text{-}gNB◃{\left\{TID_{1},TS_{1},n_{1}\cdot p,Sig_{1}\right\}}_{PK^{-1}}$

**Messages-5**: The s-gNB sends $\left\{TID_{1},n_{1}\cdot p,Sig_{1}\right\}$ to AMF.

(M5) $AMF◃{\left\{TID_{1},n_{1}\cdot p,Sig_{1}\right\}}_{PK^{-1}}$

**Messages-6**: The AMF sends $\left\{GID,TS_{1},(TID_{1}||\ldots ||TID_{i}),n_{1}\cdot p,Sig_{1}\right\}$ to t-gNB.

(M6) $t\text{-}gNB◃{\left\{GID,TS_{1},(TID_{1}||\ldots ||TID_{i}),n_{1}\cdot p,Sig_{1}\right\}}_{PK^{-1}}$

**Messages-7**: The t-gNB sends $\left\{TID_{t},GID_{1},TS_{t},n_{t}\cdot p,Sig_{t}\right\}$ to $MTCD_{1}$.

(M7) $MTCD_{1}◃{\left\{TID_{t},GID_{1},TS_{t},n_{t}\cdot p,Sig_{t}\right\}}_{GID}$

**Messages-8**: The $MTCD_{1}$ sends $\left\{GID_{1},TID_{t},TID_{1},TS_{1}\right\}$ to $MTCD_{i}.$

(M8) $MTCD_{i}◃{\left\{GID_{1},TID_{t},TID_{1},TS_{1}\right\}}_{GID}$

**Messages-9**: The $MTCD_{i}$ sends $\left\{TID_{i},TS_{i},n_{i}\cdot p,Sig_{i}\right\}$ to $MTCD_{1}$.

(M9) $MTCD_{1}◃{\left\{TID_{i},TS_{i},n_{i}\cdot p,Sig_{i}\right\}}_{PK^{-1}}$

**Messages-10**: The $MTCD_{1}$ sends $\left\{(TID_{2}||\ldots ||TID_{i}),n_{2}\cdot p||\ldots ||n_{i}p,Sig,TS_{i}\right\}$ to t-gNB.

(M10) $t\text{-}gNB◃{\left\{(TID_{2}||\ldots ||TID_{i}),n_{2}\cdot p||\ldots ||n_{i}\cdot p,Sig,TS_{i}\right\}}_{PK^{-1}}$

**Messages-11**: The t-gNB sends $\left\{TID_{t},TS_{t},n_{t}\cdot p,Sig_{t}\right\}$ to $MTCD_{1}$.

(M11) $MTCD_{1}◃{\left\{TID_{t},TS_{t},n_{t}\cdot p,Sig_{t}\right\}}_{GID}$

**Messages-12**: The $MTCD_{1}$ sends $\left\{TID_{t},TS_{1},TS_{t},n_{t}\cdot p,Sig_{t}\right\}$ to $MTCD_{i}$.

(M12) $MTCD_{i}◃{\left\{TID_{t},TS_{1},TS_{t},n_{t}\cdot p,Sig_{t}\right\}}_{GID}$

We refer to all MTC devices, including MTCD and $MTCD_{i}$, as MTCD.

#### 6.1.2. Logical Assumptions

The initial assumptions for protocol analysis are as follows: MTCDs and t-gNB trust locally generated random numbers, as well as the key pairs generated from these random numbers. The random number n includes both $n_{1}$ and $n_{i}$, and the MTCDs include both $MTCD_{1}$ and $MTCD_{i}$.

(A1) $MTCD_{1}\left|\equiv \right.n_{1}$, $MTCD_{i}|\equiv n_{i}$

(A2) $MTCD_{1}|\equiv n_{1}\cdot p$, $MTCD_{i}|\equiv n_{i}\cdot p$

(A3) $t\text{-}gNB|\equiv n_{t}$

(A4) $t\text{-}gNB|\equiv n_{t}\cdot p$

Both MTCDs and t-gNB, upon receiving messages, verify the timestamps. Therefore, they trust the freshness of the timestamps.

(A5) $MTCD|\equiv \#(TS_{t})$

(A6) $t\text{-}gNB|\equiv \#(TS_{1})$, $t\text{-}gNB|\equiv \#(TS_{i})$

MTCDs should trust that the keys generated by t-gNB are under its control and trustworthy. Similarly, t-gNB should trust that the keys generated by $MTCD_{i}$ are under its control and trustworthy. This mutual trust is established because $MTCD_{i}$ undergoes the initial handover authentication process. And upon successful authentication, it gains complete trust from t-gNB. Consequently, $MTCD_{i}$ should also trust that the keys from t-gNB received by $MTCD_{i}$ are under control and trustworthy.

(A7) $MTCD|\equiv t\text{-}gNB\Rightarrow n_{t}\cdot p$

(A8) $t\text{-}gNB|\equiv MTCD_{1}\Rightarrow n_{1}\cdot p$, $t\text{-}gNB|\equiv MTCD_{i}\Rightarrow n_{i}\cdot p$

(A9) $MTCD\left|\equiv t\text{-}gNB\right|\equiv n_{t}$

(A10) $t\text{-}gNB\left|\equiv MTCD_{1}\right|\equiv n_{1}$, $t\text{-}gNB\left|\equiv MTCD_{i}\right|\equiv n_{i}$

Both t-gNB and MTCDs possess the same group key. The group key is generated by MTCDs in the handover process and then sent to t-gNB. Both parties trust this key.

(A11) $MTCD|\equiv MTCD\overset{GID}{⟷}t\text{-}gNB$

(A12) $t\text{-}gNB|\equiv \overset{PK}{\to }MTCD$

The group key GID is the shared key among the group of MTCDs.

#### 6.1.3. Protocol Goal

The purpose of the handover authentication is to accomplish mutual authentication and key agreement between each MTCD and the t-gNB. Since the first $MTCD_{1}$ and subsequent $MTCD_{i}$ have different authentication processes, we will prove them separately. In the model, all MTCDs, including $MTCD_{1}$ and $MTCD_{i}$, are represented as MTCD, and the timestamp is denoted as TS. The specific objectives are described as follows:

G1–G8 are objectives. If all 8 objectives are achieved, then the session key, which is known only to MTCD and t-gNB, will be shared between them.

(G1) $MTCD_{1}|\equiv MTCD_{1}\overset{K_{1t}}{⟷}t\text{-}gNB$

(G2) $t\text{-}gNB|\equiv t\text{-}gNB\overset{K_{1t}}{⟷}MTCD_{1}$

(G3) $MTCD_{1}\left|\equiv t\text{-}gNB\right|\equiv t\text{-}gNB\overset{K_{1t}}{⟷}MTCD_{1}$

(G4) $t\text{-}gNB\left|\equiv MTCD_{1}\right|\equiv MTCD_{1}\overset{K_{1t}}{⟷}t\text{-}gNB$

(G5) $MTCD_{i}|\equiv MTCD_{i}\overset{K_{it}}{⟷}t\text{-}gNB$

(G6) $t\text{-}gNB|\equiv t\text{-}gNB\overset{K_{it}}{⟷}MTCD_{i}$

(G7) $MTCD_{i}\left|\equiv t\text{-}gNB\right|\equiv t\text{-}gNB\overset{K_{it}}{⟷}MTCD_{i}$

(G8) $t\text{-}gNB\left|\equiv MTCD_{i}\right|\equiv MTCD_{i}\overset{K_{it}}{⟷}t\text{-}gNB$

#### 6.1.4. Protocol Verification

Using the rules, assumptions, and messages, the detailed proof is as follows:

According to R1 and considering A12 and M6, we can deduce

(2) $$t\text{-}gNB|\equiv MTCD_{1}∼\{GID,TS_{1},(TID_{1}||\ldots ||TID_{i}),n_{1}\cdot p,Sig_{1}\}$$

According to R2 and considering A6, we can deduce

(3) $$t\text{-}gNB|\equiv \#\{GID,TS_{1},(TID_{1}||\ldots ||TID_{i}),n_{1}\cdot p,Sig_{1}\}$$

According to R3 and considering (2) and (3), we can deduce

(4) $$t\text{-}gNB|\equiv MTCD_{1}|\equiv \{GID,TS_{1},(TID_{1}||\ldots ||TID_{i}),n_{1}\cdot p,Sig_{1}\}$$

According to R4 and considering (4), we can deduce

(5) $$t\text{-}gNB|\equiv MTCD_{1}|\equiv \{n_{1}\cdot p\}$$

According to R5, along with A8 and (5), we can deduce

(6) $$t\text{-}gNB|\equiv \{n_{1}\cdot p\}$$

According to the R1 and considering A11 and M7, we can deduce

(7) $$MTCD_{1}|\equiv t\text{-}gNB∼\{GID_{1},TS_{t},n_{t}\cdot p,Sig_{t}\}$$

According to R2 and considering A5, we can deduce

(8) $$MTCD_{1}|\equiv \#\{GID_{1},TS_{t},n_{t}\cdot p,Sig_{t}\}$$

According to R3 and considering (7) and (8), we can deduce

(9) $$MTCD_{1}|\equiv t\text{-}gNB|\equiv \{GID_{1},TS_{t},n_{t}\cdot p,Sig_{t}\}$$

According to R4 and considering (9), we can deduce

(10) $$MTCD_{1}|\equiv t\text{-}gNB|\equiv \{n_{t}\cdot p\}$$

According to R5 and considering (10) and A7, we can deduce

(11) $$MTCD_{1}|\equiv \{n_{t}\cdot p\}$$

According to R4 and considering (6) and A3, we can deduce

(12) $$t\text{-}gNB|\equiv \{n_{1}\cdot n_{t}\cdot p\}$$

Since $n_{1}\cdot n_{t}\cdot p$ is the shared key $K_{1t}$, between ${MTCD}_{1}$ and t-gNB; therefore, according to Equation (12), we can deduce:

(13) $$t\text{-}gNB|\equiv t\text{-}gNB\overset{K_{1t}}{⟷}MTCD_{1}$$

According to R4 and considering (11) and A1, we can deduce

(14) $$MTCD_{1}|\equiv \{n_{1}\cdot n_{t}\cdot p\}$$

As mentioned above, according to (14), we can deduce

(15) $$MTCD_{1}|\equiv MTCD_{1}\overset{K_{1t}}{⟷}t\text{-}gNB$$

Furthermore, to complete the process, t-gNB receives M6 and verifies it. It must trust M6 to proceed with the protocol and send M7. Therefore, if $MTCD_{1}$ has already received M7, $MTCD_{1}$ can infer that t-gNB already trusts M6. $n_{1}\cdot p$ is included in M6. Therefore, we can deduce

(16) $$MTCD_{1}|\equiv t\text{-}gNB|\equiv \{n_{1}\cdot p\}$$

According to R4 and considering (16) and A9, we can deduce

(17) $$MTCD_{1}|\equiv t\text{-}gNB|\equiv MTCD_{1}\overset{K_{1t}}{⟷}t\text{-}gNB$$

As mentioned above, according to M7 and M8, we can deduce

(18) $$t\text{-}gNB|\equiv MTCD_{1}|\equiv \{n_{t}\cdot p\}$$

According to R4 and considering (18) and A10, we can deduce

(19) $$t\text{-}gNB|\equiv MTCD_{1}|\equiv t\text{-}gNB\overset{K_{1t}}{⟷}MTCD_{1}$$

According to R1 and considering M10 and A12, we can deduce

(20) $$t\text{-}gNB|\equiv MTCD_{i}∼\{(TID_{2}||\ldots ||TID_{i}),n_{2}\cdot p||\ldots ||n_{i}\cdot p,Sig,TS_{i}\}$$

According to R2 and considering A6, we can deduce

(21) $$t\text{-}gNB|\equiv \#\{(TID_{2}||\ldots ||TID_{i}),n_{2}\cdot p||\ldots ||n_{i}\cdot p,Sig,TS_{i}\}$$

According to R3 and considering (20) and (21), we can deduce

(22) $$t\text{-}gNB|\equiv MTCD_{i}|\equiv \{(TID_{2}||\ldots ||TID_{i}),n_{2}\cdot p||\ldots ||n_{i}\cdot p,Sig,TS_{i}\}$$

According to R4 and considering (22), we can deduce

(23) $$t\text{-}gNB|\equiv MTCD_{i}|\equiv \{n_{i}\cdot p\}$$

According to R5 and considering (23) and A8, we can deduce

(24) $$t\text{-}gNB|\equiv \{n_{i}\cdot p\}$$

According to R1 and considering M12 and A11, we can deduce

(25) $$MTCD_{i}|\equiv t\text{-}gNB∼\{TS_{1},TS_{t},n_{t}\cdot p,Sig_{t}\}$$

According to R2 and considering A5, we can deduce

(26) $$MTCD_{i}|\equiv \#\{n_{t}\cdot p,TS_{1},Sig_{t}\}$$

According to R3 and considering (25) and (26), we can deduce

(27) $$MTCD_{i}|\equiv t\text{-}gNB|\equiv \{n_{t}\cdot p,TS_{1},Sig_{t}\}$$

According to R4 and considering (27), we can deduce

(28) $$MTCD_{i}\equiv t\text{-}gNB|\equiv \{n_{t}\cdot p\}$$

According to R5 and considering (28) and A7, we can deduce

(29) $$MTCD_{i}|\equiv \{n_{t}\cdot p\}$$

According to R4 and considering (29) and A1, we can deduce

(30) $$MTCD_{i}|\equiv \{n_{i}\cdot n_{t}\cdot p\}$$

As mentioned above, according to (30), we can deduce

(31) $$MTCD_{i}|\equiv MTCD_{i}\overset{K_{it}}{⟷}t\text{-}gNB$$

According to R4 and considering (31) and A3, we can deduce

(32) $$t\text{-}gNB|\equiv \{n_{i}\cdot n_{t}\cdot p\}$$

As mentioned above, according to (32), we can deduce

(33) $$t\text{-}gNB|\equiv t\text{-}gNB\overset{K_{it}}{⟷}MTCD_{i}$$

Similar to $MTCD_{1}$, t-gNB also receives M10 first, and it must trust M10 in order to proceed with the protocol and send M12. If $MTCD_{i}$ has already received M12, then $MTCD_{i}$ can conclude that t-gNB now trusts M10. According to M10 and M12, we can deduce

(34) $$MTCD_{i}|\equiv t\text{-}gNB|\equiv \{n_{i}\cdot p\}$$

According to R4 and considering (34) and A9, we can deduce

(35) $$MTCD_{i}|\equiv t\text{-}gNB|\equiv MTCD_{i}\overset{K_{it}}{⟷}t\text{-}gNB$$

Likewise, based on M12, after $MTCD_{i}$ verifies the identity of t-gNB without errors, it generates a session key for subsequent communication. From this, we can deduce

(36) $$t\text{-}gNB|\equiv MTCD_{i}|\equiv \{n_{t}\cdot p\}$$

According to R4 and considering (36) and A10, we can deduce

(37) $$t\text{-}gNB|\equiv MTCD_{i}|\equiv t\text{-}gNB\overset{K_{it}}{⟷}MTCD_{i}$$

In summary, we have achieved all the security objectives, ensuring key negotiation and mutual authentication in the protocol. Our protocol has been logically validated.

### 6.2. Formal Verification

Scyther Tool is a formal verification tool commonly used for validating security protocols [26]. Scyther offers four security statements to ensure protocol consistency and detect various attacks like message forgery, replay, and man-in-the-middle (MITM). These include “Aliveness” for completing protocol steps with active responders, “Niagree” for the correct variable receipt without one-to-one communication, “Nisynch” for the expected protocol operation without one-to-one synchronization, and “Weakagree” for one-to-one communication within the same group of initiators or responders.

**Figure 6:**
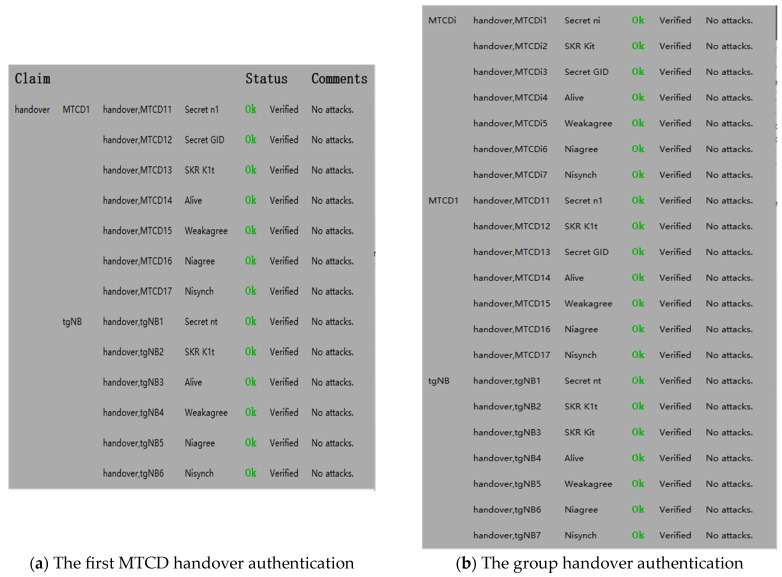
Results of formal verification.

The verification results are shown in Figure 6, where Figure 6a demonstrates the first MTCD handover authentication phase, and Figure 6b demonstrates the group handover authentication phase. Specifically, the model is created with three roles: t-gNB, $MTCD_{i}$, and $MTCD_{1}$. Initially, the BSPGH scheme achieves mutual key agreement using the Elliptic Curve Diffie–Hellman (ECDH). To simulate the ECDH key exchange between two parties, two functions are defined as g1 and g2. Then, $n_{1}\cdot P$ is set to g1(n1), $n_{i}\cdot P$ is set to g1(ni), and $n_{t}\cdot P$ is set to g1(nt). The confidentiality of the ECDH private keys is first verified using the declarations of Secret n1, Secret ni, and Secret nt. Next, the derivation of ECDH public keys is performed using K1t (i.e., g2(nt, g1(n1))) and Kit (i.e., g2(nt, g1(ni))). The confidentiality of the ECDH private keys is ensured by declaring Secret ni, Secret nt, and Secret n1. Additionally, the confidentiality of the ECDH public key is validated by the SKR declaration. Our protocol assumes that $MTCD_{i}$ needs to use the group key GID to verify the identity of $MTCD_{1}$. To ensure that adversaries cannot obtain the group key GID in any way, its secrecy is checked using the Secret GID declaration. Finally, the verification results reveal that all participants in the system satisfy the properties of synchronous (Nisynch), consistent (Niagree), active (Alive), and weak consistency (Weakagree). All the keys meet the security requirements. Therefore, our protocol is deemed secure by the verification using Scyther.

### 6.3. Security Analysis

- **Mutual Authentication:** By the BSPGH scheme, a gNB verifies the authenticity of the MTCD’s signature Sig by retrieving the public key stored on the blockchain, thereby confirming the identity of the MTCD. Since digital signatures are generated by encrypting messages with a private key and it is computationally infeasible to deduce the private key from the public key, this way allows the MTCD to effectively prove the legitimacy of its authentication request to the gNB. Furthermore, the gNB employs hash-based message authentication code (HMAC) scheme and uses the group key GID as the key for the HMAC to generate a signature ${Sig}_{t}$. The MTCD, possessing a legitimate GID, verifies ${\;Sig}_{t}$ to prove the legitimacy of the response. In this way, the MTCD and the gNB are able to achieve mutual authentication, ensuring the security of the communication between them.
- **Privacy protection:** The temporary identity identifier $TID_{i}$ is transmitted to each participant over the wireless channel, ensuring that the real identity is only disclosed to legitimate gNBs and the core network. It satisfies anonymity requirements.
- **Perfect forward secrecy and backward secrecy:** By the proposed protocol, the generation of the session key $K_{it}$ relies on randomly generated ECDH parameters. Without the session key, attackers cannot recover the contents of a specific session. Moreover, since a session key is generated for each session and the ECDH parameters for each session key are independent of those from previous or future sessions, the leakage of a session key would only affect the current session. The confidentiality of previous or future sessions would remain unaffected.
- **Replay and impersonation attacks resistance:** By the proposed protocol, authentication requests and responses are both marked with a timestamp TS. The TS constraint ensures that messages are received within a specified time window, allowing easy identification. The replayed messages will be discarded, thus, countering replay attacks. The use of a key system based on discrete logarithms makes deriving private keys from the public keys challenging, preventing attackers from forging signatures and thus impersonating legitimate identities. It can effectively strengthen the ability of the protocol to resist impersonation attacks.
- **DoS/DDoS attacks resistance:** The receiving gNB first verifies the timestamp’s validity and then compares the signature with records in the blockchain for authentication. If they do not match, the session is immediately terminated, preventing attackers from consuming gNB’s computational resources through replay attacks. The failure of one gNB or one AMF will not affect the entire 5G wireless network. Therefore, it can prevent DDoS attacks in 5G authentication.
- **Session key leakage:** Attackers may attempt to compute the session key to steal messages transmitted over the wireless channel. However, the session key is formed based on ECDH by the proposed scheme, which relies on the difficulty of the elliptic curve discrete logarithm problem. Attackers cannot obtain $n_{i}$ and $n_{t}$ from $n_{i}\cdot p$ and $n_{t}\cdot p$, and thus cannot compute the session key $n_{i}\cdot n_{t}\cdot p$. It effectively prevents session key leakage.
- **Sybil attack:** By the proposed protocol, a private blockchain is utilized. Within a private blockchain, access and participation in the network are restricted, allowing only MTCDs that have been registered by core network entities to join and interact. Furthermore, the identities of MTCDs must be verified subsequently, which limits the ability of attackers to forge numerous identities to conduct attacks. Therefore, this approach is capable of resisting Sybil attacks, where an attacker creates a large number of pseudonymous identities to compromise the network.
- **Man-in-the-middle attack:** By the BSPGH scheme, an adversary cannot impersonate a legitimate t-gNB to deceive an MTCD because a temporary session key $K_{\mathrm{it}}$ is established between them using the ECDH. The adversary cannot obtain or modify the temporary session key; thus, it is unable to establish communication with the MTCD.
- **Linkability attack prevention:** In the BSPGH authentication process, the PK and TID are periodically updated, while the elements in other messages are random numbers. BSPGH employs ECDH for session key generation instead of using serial numbers, which prevents the common MAC failures or synchronization issues found in symmetric key-based AKA protocols. This approach makes it difficult for attackers to analyze the correlation between different messages or to exploit erroneous messages as vulnerabilities to track a specific device. Consequently, BSPGH effectively safeguards against linkability attacks, enhancing privacy and security in the communication process.

## 7. Performance Evaluation

In this section, we evaluated the performance of the BSPGH scheme in terms of computational cost and communication cost during the first handover authentication and group handover authentication phases and compare its performance with those of three other protocols, namely, 5G standard specified by 3GPP TS 23.501 R16 [21], a privacy-preserving handover authentication protocol for a group of MTC devices in 5G networks (PPHAP) [1], and a novel authentication scheme supporting multiple user access for 5G and beyond (NASS) [7]. In the analysis, it is assumed that all symmetric encryption keys are 256 bits, MACs are 160 bits, and elements in the Hash functions, TID, GID, Sig, n·P, and $Z_{q}^{*}$ are 128 bits. The timestamp is represented by 32 bits, and elements in group G have sizes of 320 bits.

### 7.1. Blockchain Operation Cost

To evaluate the data access latency in the blockchain, we followed the evaluation methodology outlined in [27]. Initially, a blockchain prototype was created. This blockchain comprised numerous blocks, each being 1 MB in size. Every block, stored as a file, contained multiple transaction records, with each transaction being a subscription record of a device. To reduce the storage overhead of the blockchain, this size should be adjusted according to the preferences of the network operators. By default, we followed the Bitcoin model with a block size of 1 MB. All transactions in the blockchain were indexed using Python dictionaries. To compare the performance differences between the blockchain and traditional databases, another centralized database was also constructed using MariaDB v10.4.14 [28]. The read operation $T_{BC.read}$ is 0.2914 ms and the write operation $T_{BC.write}$ is 0.0434 ms, while for the centralized database, both of the read and write operations $T_{DB}$ are 0.4956 ms.

By the proposed scheme, since the writing and querying operations of the blockchain information occur prior to the two phases of handover authentication, specifically during the registration phase and the handover preparation phase, the time taken for blockchain operations has not been included in the computational costs of the first handover authentication and group handover authentication phases.

### 7.2. Computational Cost

We utilize a device equipped with an Intel(R) Core (TM) i7-12700H processor running at 3.50 GHz and 16 GB of RAMs for the evaluation of the computational cost of the two phases of handover authentication. The cryptographic library employed to perform the required cryptographic operations of the proposed scheme was C/C++ OPENSSL. For the selected protocols, each protocol uses different encryption functions with unique key size requirements. To assess their performance comprehensively and fairly at the same security level, we follow the recommendation of NIST in [28] and use a 256-bit equivalent key strength throughout the entire simulation process. Therefore, for all operations based on ECC, secp256k1 is selected as the default elliptic curve. We run each encryption function 10,000 times to measure its average execution time. The measurement results obtained follow. The point multiplication $T_{\mathrm{PM}}$ is 0.201 ms. The point addition $T_{\mathrm{PA}}$ is 0.001 ms. The modular exponentiation $T_{E}$ is 0.665 ms. The Rivest–Shamir–Adleman (RSA) signature and verification $T_{\mathrm{RV}}$ is 1.445 ms. The hash operation $T_{H}$ is 0.021 ms. The symmetric encryption/decryption operation $T_{A}$ is 0.02 ms. XOR, multiplication, and arithmetic operations have been neglected. The results are presented in Table 2, where $T_{\mathrm{MTCD}}$ and $T_{\mathrm{gNB}}$ represent the computation time for the MTCD and the gNB, respectively. We have only accounted for the operations during the first MTCD handover authentication and the group handover authentication of the BSPGH scheme.

In Table 2, “*n*” represents the number of MTCD to substitute specific numerical values. For the 5G-AKA scheme, the computation costs are 0.126n ms. For the PPHAP scheme, the computation costs are 0.205n-0.063 ms. For the NASS scheme, the computation costs are 1.466n+2.775 ms. For the BSPGH scheme, the computation costs are 1.155n+1.068 ms. The NASS scheme involves complete RSA verification for handover authentication, which increases the overhead. The PPHAP scheme primarily uses symmetric encryption and hash operations for authentication, resulting in lower computational overhead but posing a risk of DDoS attacks. The 5G-AKA scheme has the lowest computational overhead but has serious security vulnerabilities. In contrast, the proposed BSPGH scheme is the most secure in terms of security functionality and has lower overhead compared to the NASS scheme. Overall, the BSPGH scheme has been proven to be the most effective.

### 7.3. Communication Cost

The communication costs for *n* MTCDs to perform handover authentication by the BSPGH scheme are evaluated and compared with the other three protocols. The communication costs include propagation time and transmission time. The propagation time is determined by the distance between the transmitter and the receiver and the propagation speed over the wireless communication channel, which is approximately $3\times 10^{8}$ m/s. It is assumed that the radius of a cell is 200 m, and the data packets sent by an MTCD would take 200 m to propagate to the gNB at a speed of $3\times 10^{8}$ m/s. According to the 3GPP standard on 5G communication, the downlink data rate for the urban general area scenario is 50 Mbps, and the uplink data rate is 25 Mbps [29]. In Table 3, “Amount of Information” refers to the total amount of data transmitted during the communication process. $T_{t}$ and $T_{p}$ represent transmission delay and propagation delay, respectively. “Up” and “Down” denote uplink and downlink data transmission. Therefore, we compare them separately. When calculating communication costs, we consider only the authentication phases during the handover, including the first MTCD handover authentication, group handover authentication.

**Figure 7:**
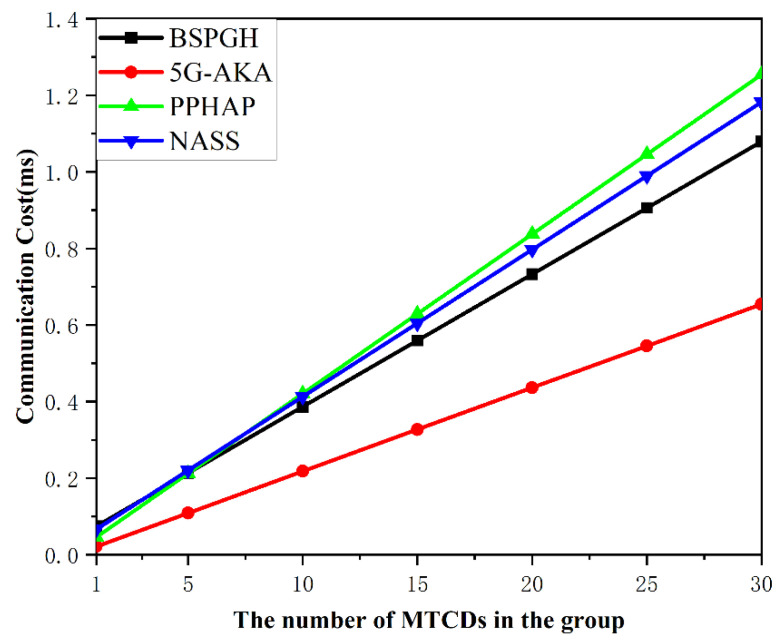
Comparison of communication cost.

Figure 7 demonstrates that when there is a large number of group MTCDs, the proposed BSPGH scheme exhibits superior performance in terms of communication overhead compared to the PPHAP and the NASS scheme. This advantage is attributed to the reduced information exchanged between MTCDs and gNBs in our protocol, leading to enhanced efficiency. In contrast, the communication overhead of the 5G-AKA scheme is lower, but it suffers from a lot of security vulnerabilities. Overall, the results indicate the better performance achieved by the BSPGH scheme.

### 7.4. Authentication Cost

**Figure 8:**
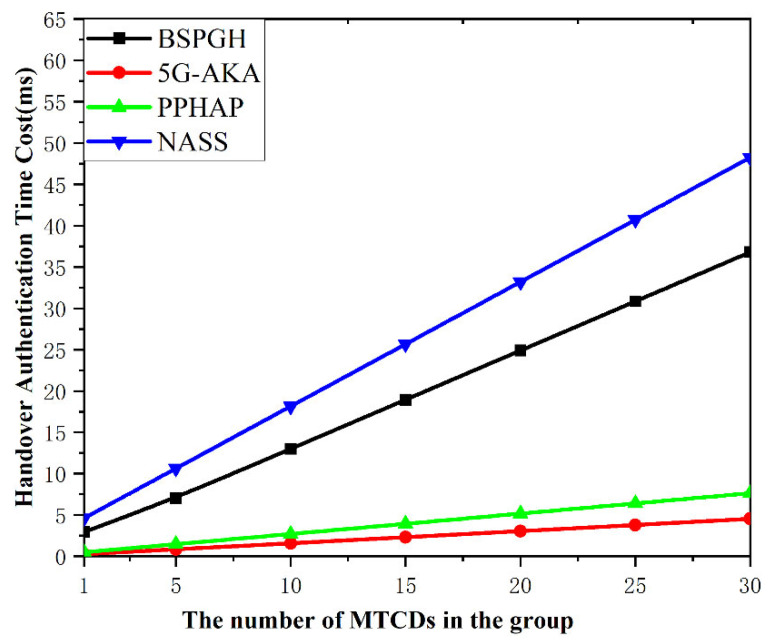
Comparison of the authentication cost.

Figure 8 shows the total time cost of the handover authentications for n MTCDs within a group. The comparison of four handover authentication schemes is as follows. The NASS scheme needs a far higher handover authentication cost compared to the BSPGH scheme, due to the involvement of complete RSA signature verification and modular exponentiation for computation and a higher communication cost during the authentication process; thus, it increases the overall latency. The PPHAP scheme has a lower handover authentication time cost, while the BSPGH scheme provides enhanced security features in mitigating DDoS attacks. The 5G-AKA scheme has a lower latency but suffers from serious security vulnerabilities. Therefore, although the BSPGH protocol inevitably introduces a minor computational overhead, it remains the most effective protocol in balancing the security functionality and system performance.

We assess the robustness of the protocol for estimating the robustness of handover authentication. In the system, handover authentication may be forced to stop and restart when facing unknown attacks. We assume that unknown attacks can occur at each step of the handover authentication, and the probability of unknown attacks is uniform [1]. The average time of successful handover authentication is calculated as follows:

(38) $$T=\frac{T_{success}+T_{failed}}{N_{success}}=\frac{\sum_{i=1}^{n}\frac{1}{n}\times t_{fail}\times p+t_{success}\times (1-p)}{1-p}$$

where T, $T_{success}$, and $T_{failed}$ are the average time taken for a successful handover authentication, the total time of successful handover authentications, and the total time for failed handover authentications, respectively, $N_{success}$ is the number of successful handover authentications, p is the percentage of unknown attacks, n is the number of steps in the protocol, $t_{\mathrm{fail}}$ represents the total time cost before the attack happens in the *i*-th step, and $t_{\mathrm{success}}$ represents the time elapsed for one successful handover authentication before an attack occurs.

**Figure 9:**
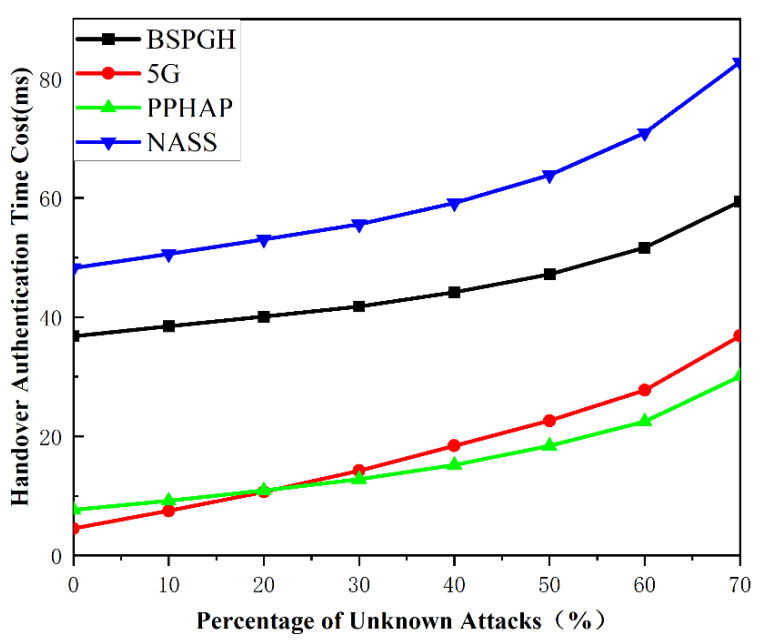
Comparison of the authentication cost for 30 MTCDs with unknown attacks.

The simulation results of comparison with a group size of 30 MTCDs are shown as in Figure 9, when the percentage of unknown attacks increases. It is evident that the BSPGH protocol has a lower total time cost compared to NASS, indicating a better performance. However, it has a higher time cost compared to the solutions of the 5G standard and the PPHAP scheme. This is because that the 5G standard and the PPHAP scheme use a symmetric cryptographic system and have suffered security vulnerabilities. As shown in Figure 6, our proposed protocol can resist most major malicious attacks, albeit with a slightly higher total time cost. Overall, the proposed BSPGH scheme demonstrates a better performance in terms of security functionality and system efficiency.

### 7.5. Energy Consumption

The security protocols that offer the highest level of security functionality while consuming the least amount of energy are always the preferred choice due to the limited battery life of mobile devices like MTCDs. To evaluate the energy consumption of MTCDs, two factors should be considered including the energy required for data transmission and the energy consumed for the execution of the cryptographic functions. For the energy used for data transmission, the evaluation of energy consumption follows the data transmission power model described in [28]. The calculation of the transmission energy cost for the uplink and downlink is as follows:

(39) $$E_{ul}=(\alpha_{u}t_{udr}+\beta )\cdot t_{ul}$$

(40) $$E_{dl}=(\alpha_{d}t_{ddr}+\beta )\cdot t_{dl}$$

where $\alpha_{u}=438.39\;\mathrm{mW}/\mathrm{Mbps}$, $\alpha_{d}=51.97\;\mathrm{mW}/\mathrm{Mbps}$, β=1288.04 mW, $t_{udr}=25\;\mathrm{Mbps}$, and $t_{ddr}=50\;\mathrm{Mbps}$. $t_{udr}$ represents the uplink throughput, $t_{ddr}$ represents the downlink throughput, $t_{ul}$ is the transmission time for uplink, and $t_{dl}$ is the transmission time for downlink.

For the energy used for performing cryptographic functions, the way of approximation of energy cost in [30] is adopted. All experiments were conducted using a battery-powered Compaq iPAQ H3670 PDA, which is equipped with an Intel SA-1110 StrongARM processor running at 206 MHz, 64 MB RAM, and 16 MB FlashROM for evaluation. The energy cost of a single AES encryption or decryption operation is 7.87+1.21b μJ, where b is the number of bytes in the plaintext. The energy cost per byte for a SHA-1 hash operation $E_{H}$ is 0.76 μJ. Considering that the energy cost for generating an ECDH public key is 276.7 mJ and for deriving an ECDH public key is 163.5 mJ, the energy consumption for a single scalar multiplication operation $E_{PM}$ is approximately estimated to be 220.1 mJ, and the energy cost for a single RSA operation $E_{RV}$ is 832.6 mJ. The energy consumption of using cryptographic primitives is derived based on the cryptographic operations used in the protocol, leading to a theoretical energy cost.

**Figure 10:**
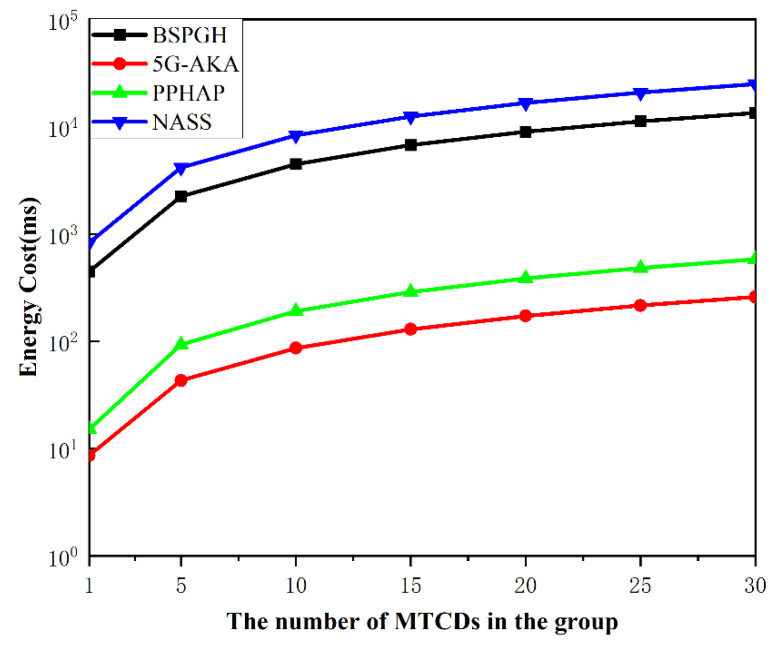
Comparison of the energy consumption.

Figure 10 illustrates the relationship between the energy consumption during handover authentication and the number of MTCDs in a group. It is clearly visible from the figure that, compared to the NASS protocol, the energy consumption of BSPGH is lower and more efficient as the number of MTCDs increases. Additionally, the energy consumption of the 5G and PPHAP protocols is lower than our protocol. However, the 5G standard protocol has security issues and is susceptible to different malicious attacks, while the PPHAP protocol is also vulnerable to security threats like DDoS attacks. On the other hand, the BSPGH protocol can significantly enhance network security, albeit at a slightly higher cost compared to other protocols. It can also facilitate group handover authentication for a large number of users simultaneously. Therefore, the BSPGH protocol exhibits a better performance in terms of both security and efficiency.

### 7.6. Discussion of the Simulation Results

We have Table 4 to discuss the security features of the BSPGH, NASS, PPHAP, and 5G-AKA schemes in detail. The 5G-AKA scheme faces security problems in handover authentication including lack of forward secrecy for keys, vulnerability to DoS and DDoS attacks, session key leakage, susceptibility to linkability attacks, and the absence of mutual authentication between an MTC device and its target gNB. The NASS scheme is vulnerable to DoS and DdoS attacks. The PPHAP scheme struggles to resist DdoS attacks. On the other hand, the BSPGH scheme can overcome all of the abovementioned security vulnerabilities, ensuring the highest level of security during the handover authentication process.

We have conducted experiments for performance comparison among the four handover authentication schemes in terms of handover authentication time for a group of 30 MTC devices. Our proposed protocol, the BSPGH scheme, is faster than the NASS scheme. This time improvement comes from the fact that the NASS scheme performs a full RSA signature verification and modular exponentiation calculations, which result in significant computational overhead, while the BSPGH approach works based on certificateless aggregate signatures and ECDH for session key generation, which need only point multiplication, point addition, and hashing operations so that relatively lower computational costs are incurred. The reduction in computational functions leads to a shorter authentication time, making the BSPGH scheme faster than the NASS scheme for handover authentication.

However, the BSPGH scheme takes a longer time for handover authentication compared to the 5G-AKA and the PPHAP schemes. This is because the BSPGH scheme employs asymmetric ECDH to replace the less secure symmetric key-based key derivation function (KDF) used in the 5G-AKA and the PPHAP scheme. The computational overhead of the ECDH is much higher than that of the KDF, but the KDF is weak to resistant exhaustive attacks, side-channel attacks, and replay attacks. By incorporating the ECDH, the BSPGH scheme cannot only address the security issues associated with key derivation functions but also ensure perfect forward secrecy/backward secrecy and the prevention of linkability attacks for session keys.

Considering the exponential growth in the number of 5G mobile devices in the future, the risks associated with DoS and DDoS attacks in large-scale MTCD scenarios will become more pronounced. Our solution effectively leverages the decentralized nature of blockchain, ensuring the authenticity and security of critical information, and more efficiently defending against DoS and DDoS attacks during the multi-user authentication process. Moreover, our protocol exhibits outstanding performance in the environments susceptible to these security attacks. Therefore, we believe that these overheads are tolerable to protect future 5G wireless networks.

## 8. Conclusions

In this paper, we have proposed the blockchain-assisted group handover authentication protocol for MTC communication in 5G wireless networks. The BSPGH protocol aims to reduce the authentication time when multiple MTCDs undergo frequent handovers between gNBs by adopting group handover authentication. The proposed BSPGH scheme requires no modification to the existing 5G network architecture specified by the 3GPP standard, making it easy to deploy. By formal verification using Scyther Tool and BAN-logic, we have analyzed the protocol’s security properties, demonstrating that it can provide perfect forward secrecy and resist impersonation attacks, DoS/DDoS attacks, and some other attacks. It also ensures the anonymity of the MTCDs. Moreover, the performance analysis has shown that the BSPGH scheme can meet the requirements of various application scenarios to confirm its efficiency.

## Figures and Tables

**Table 1:** Notations and definition of the proposed protocol.

Notation	Definition
TID	Temporary anonymous identity
ID	Permanent identifier
GID	Group key material
MAC	Message authentication code
H(msg)	Hash function
TS	Timestamp
$K_{it}/K_{1t}$	The session key of MTCD and gNB
G	Cyclic additive group
P/q	Generator of the group/prime order of group
PK/SK	Public key/private key
$\{x{\}}_{k}$	Encrypted x with key k

**Table 2:** Computational costs of different protocols.

Protocol	$T_{MTCD}$	$T_{gNB}$	$T_{MTCD}$(μs)	$T_{gNB}$(μs)
5G-AKA	$4nT_{H}$	$2nT_{H}$	0.084n	0.042n
PPHAP	$3n(T_{A}+T_{H})\text{-}$ $2T_{H}$	( $2T_{H}+$ $3T_{A})\;$ $n\text{-}T_{H}$	0.123n − 0.042	0.102n − 0.021
NASS	$nT_{RV}$	$nT_{H}+$ $2T_{E}+T_{RV}$	1.445n	0.021n + 2.775
BSPGH	n( $4T_{H}+$ $2T_{PM})$	$(3n+3)T_{H}+$ $(3n+5)T_{PM}+$ $3nT_{PA}$	0.486n	0.669n + 1.068

**Table 3:** Communication costs of different protocols.

Protocol	Link	Amount of Information (bits)	T_t_ (μs)	T_p_ (μs)
5G-AKA	Up	128n	5.12n	0.67n
	Down	768n	15.36n	0.67n
PPHAP	Up	608n − 160	24.32n − 6.4	0.34n + 1.068
	Down	800n + 448	16n + 8.96	1.01n + 0.34
NASS	Up	512n + 288	20.48n + 11.52	0.67n
	Down	832n + 832	16.64n + 16.64	0.67n
BSPGH	Up	768n + 64	30.72n + 2.56	0.67(n + 1)
	Down	128n + 1824	2.56n + 36.48	0.67(n + 1)

**Table 4:** Security analysis of protocols.

Protocol	Security Features									
	MA	PP	PFS	RA	IA	DoS	DDoS	SKL	SA	MITM
BSPGH	√	√	√	√	√	√	√	√	√	√
5G-AKA		√		√	√				√	√
PPHAP	√	√	√	√	√	√		√	√	√
NASS	√	√	√	√	√			√	√	√

MA = mutual authentication; PP = privacy protection; PFS = perfect forward secrecy; RA = replay attack prevention; IA = impersonation attack prevention; DoS = DoS attack prevention; DDoS = DDoS attack prevention; SKL = session key leakage; SA = sybil attack prevention; MITM = man-in-the-middle attack prevention.

## Data Availability

Data are contained within the article.
